# Re-pancreaticojejunostomy for Necrosis of the Roux-en-Y Limb Tip 14 Years After Partington-Rochelle Procedure

**DOI:** 10.7759/cureus.18142

**Published:** 2021-09-20

**Authors:** Akinori Sekioka, Shuichi Ota, Tetsuo Ito, Yo Mizukami, Yukito Adachi

**Affiliations:** 1 Gastroenterological Surgery, Osaka Saiseikai-Noe Hospital, Osaka, JPN

**Keywords:** roux-en-y limb, complication, chronic pancreatitis, partington-rochelle procedure, pancreaticojejunostomy

## Abstract

Longitudinal pancreaticojejunostomy for chronic pancreatitis, the Partington-Rochelle (PR) procedure, is a good option to control pain caused by dilation of the main pancreatic duct. However, long-term complications related to anastomosis are still unclear. Here, we present a case of a 78-year-old patient with sudden necrosis of the Roux-en-Y limb tip in a PR procedure performed 14 years ago. During emergent laparotomy, we resected the necrotic limb and re-anastomosed the remaining Roux-en-Y limb to the main pancreatic duct. Postoperatively, we managed the inflammation caused by the pancreatic fistula and successfully saved the patient by long-term drainage. Although the cause of necrosis is still unclear, mild kinking and stenosis of the Roux-en-Y limb might be associated with this situation.

## Introduction

Chronic pancreatitis is a challenging situation, sometimes requiring surgical intervention to relieve the pain caused by pancreatic ductal dilation or stones. Historically, several kinds of surgical approaches have been introduced since the 1900s. Among these, the Partington-Rochelle (PR) procedure is a useful option to control the symptoms, which is a modification of the Puestow procedure [1, 2]. This approach is a longitudinal pancreaticojejunostomy, opening the dilated main pancreatic duct and anastomosing it to the side of the Roux-en-Y (R-Y) jejunum. Although some complications were reported in previous studies, such as sustained abdominal pain, diabetes, or exocrine insufficiency, the long-term outcomes of this procedure remain still unclear [3, 4].

In this report, we present a rare complication that developed 14 years after the PR procedure. We also present an effective treatment for it, which has never been reported before.

## Case presentation

A 78-year-old man (163 cm, 70 kg) was referred to the emergency department of our hospital due to severe abdominal pain, which had worsened over the last 2 days. He had abdominal pain due to chronic alcoholic pancreatitis and underwent side-to-side pancreaticojejunostomy (PR procedure) 14 years previously. After the operation, he had suspended alcohol intake and had lived without pain related to pancreatitis.

**Figure 1:**
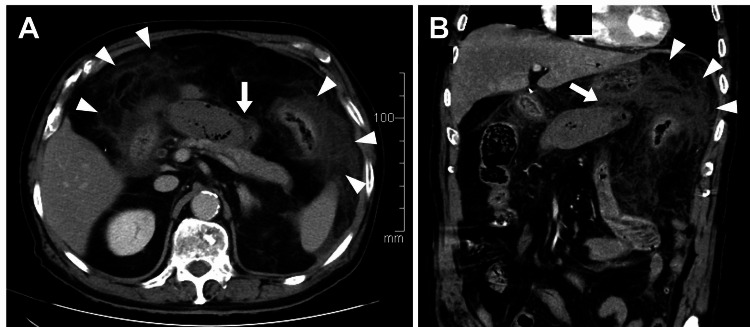
CT showing severe inflammation around the R-Y limb (A) Axial CT showing severe inflammation in the upper abdominal cavity (arrowheads), and the dilated tip of the R-Y limb (arrow). (B) Coronal CT showing severe inflammation in the left subdiaphragmatic region (arrowheads), and free air around the R-Y limb (arrow). CT, computed tomography; R-Y, Roux-en-Y

On general examination, his blood pressure was 127/81 mmHg, heart rate was 120 bpm, body temperature was 37.7 ℃, and peripheral oxygen saturation on room air was 95%. Abdominal examination showed severe upper abdominal pain and a rigid abdominal wall. Contrast-enhanced computed tomography revealed necrosis of the Roux-en-Y (R-Y) limb tip at the anastomotic site, causing perforation peritonitis (Figure 1A, 1B). Laboratory results showed inflammation and multiorgan failure: White Blood Cell count 7800/µl (stab 21%, segment 48%, metamyelocyte 17%, myelocyte 6%), C-reactive protein 20.3 mg/dL (reference range 0-0.3), Amylase 617 IU/L (reference range 39-134), Blood urea nitrogen 32.3 mg/dL (reference range 8-20), Creatinine 1.14 mg/dL (reference range 0.6-1.1), and Prothrombin Time Test international normalized ratio 1.33 (reference range 0.9-1.1). Arterial blood gas analysis showed metabolic acidosis with respiratory compensation; pH 7.422, partial pressure of oxygen and carbon dioxide 73.4 Torr and 26.1 Torr, respectively, lactate 3.6 mmol/L, bicarbonate 16.7 mmol/L, and base excess -6.0 mmol/L.

**Figure 2:**
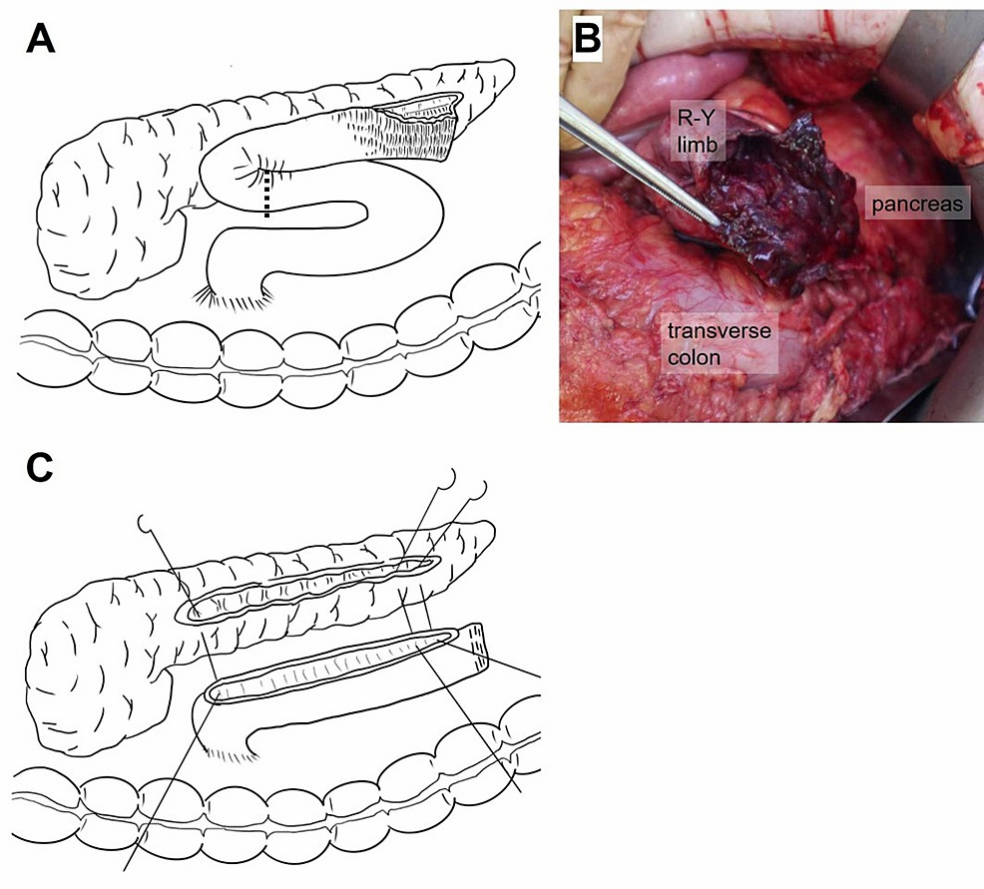
Schematic illustration and picture of the pancreaticojejunostomy (A) Schematic illustrations of the situation around the anastomotic site. The necrotic intestine was cut on the dotted line. (B)The tip of the Roux-en-Y (R-Y) limb held by forceps was already diverted from the anastomotic site with the main pancreatic duct. The tip of the R-Y limb was necrotic and perforated. (C) Schematic illustrations of the situation in the re-anastomotic procedure.

**Figure 3:**
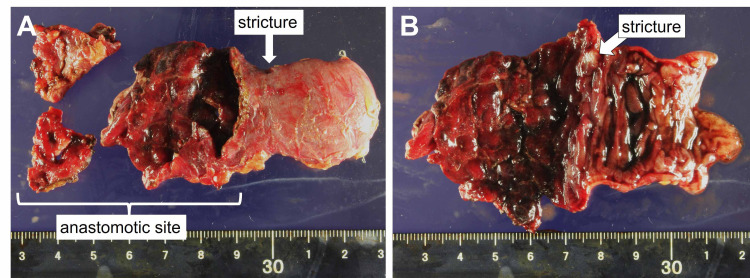
The macroscopic picture of the resected intestine (A) The macroscopic picture showing the stricture near the anastomotic site without malignancy. (B) The macroscopic picture with the opened anal side of the resected intestine.

Emergency laparotomy was performed after examination. In the upper abdominal cavity, severe adhesions were observed in the thick greater omentum, stomach, and transverse colon. After careful adhesiolysis, complete necrosis and perforation of the R-Y limb tip in the pancreaticojejunostomy site was observed. This could not be repaired by suturing (Figure 2A, 2B). We removed the necrotic jejunum, confirmed the main pancreatic duct opened for anastomosis, and then used the remaining R-Y limb to re-construct the anastomosis between the jejunum and pancreatic duct using 4-0 PDS II (Johnson & Johnson, New Jersey, USA) interrupted suture (Figure 2C). The operative time was 260 minutes, while blood loss was 1498 ml. Pathological findings included ischemic changes in the intestinal wall with mucosal cell effacement, intestinal crypt atrophy, and general tissue edema. Although luminal stricture was found near the anastomotic site, there were no malignant findings (Figure 3A, 3B). Microscopic investigations did not reveal the cause of the R-Y limb stricture.

After the operation, the patient developed a grade C postoperative pancreatic fistula (POPF) (Revised 2016 ISGPS classification and grading of POPF [5]), which was treated with continuous drainage in the intensive care unit (ICU). His general condition slowly improved, and the pancreatic fistula gradually decreased. He was discharged from the ICU on postoperative day (POD) 18 and left the hospital without abdominal symptoms on POD 251.

## Discussion

Surgical intervention for chronic pancreatitis with main pancreatic duct dilation has been widely accepted as an effective option, such as the Puestow procedure, P-R procedure, or Frey procedure [1-3, 6]. Although the short- and long-term outcomes of the procedures have been already reported in previous articles, focusing on postoperative pain control, nutritional status, or pancreatic function, there have been no reports of complications occurring 14 years after the procedure [4, 7-9]. Additionally, to the best of our knowledge, the present case about necrosis at the anastomotic site of pancreaticojejunostomy procedure has never been reported.

In this case, the pathological findings showed an abnormal stenosis in the outlet of the jejunum near the anastomotic site. This would cause the retardation of pancreatic juice, dilation of the R-Y limb tip, and subsequently congestion and necrosis at the site. Previous reports have shown that R-Y limb obstruction rarely occurs in the site passing through transverse mesocolon due to the adhesion, internal hernia, or kinking [10, 11]. Since these reports were mostly about gastric bypass surgery, it is still unclear whether this theory can be adapted to R-Y construction in pancreaticojejunostomy. As shown in Figure 2A, there was a kinking and adhesion between the R-Y limb and transverse mesocolon, which might be associated to this complication.

For distal gastrectomy or gastric bypass, antecolic R-Y reconstruction would be superior to retrocolic route in terms of short-term morbidities or long-term passage [12, 13]. However, there has been no discussion about the route of R-Y limb in the pancreaticojejunostomy procedure for chronic pancreatitis. In the present case, retrocolic route might be associated with the stricture of R-Y limb. Although surgeons take it for granted that the retrocolic route is natural for pancreaticojejunostomy for chronic pancreatitis, further study would be warranted.

The length of R-Y limb is also arguable. In the first report of PR procedure, the optimal length of R-Y limb was not specifically described, just mentioning the position and angulation of the limb [1]. In later reports, the length of R-Y limb in this procedure was 50-60 cm [3, 14]. As for the present case, the length of R-Y limb was slightly longer for the patient, which might cause mild kinking. In this context, the optimal length of R-Y limb should be decided during the operation, considering the size of the patient or the location of each organ.

A previous report suggested that the safety and efficacy of redo surgery for pancreaticojejunostomy among patients with chronic pancreatitis, however all cases underwent elective operation [15]. In the present situation, there would be a limited number of surgical options to treat the life-threatening event, only drainage or re-anastomosis. Although there was no evidence to establish a strong anastomosis in the highly inflammatory site, we chose re-anastomosis rather than placing drainage tubes only, because leaving the longitudinal opening main pancreatic duct was more likely to decrease the possibility of survival. Fortunately, the patient recovered, owing to the resection of the necrotic tissue and covering the main pancreatic duct by the jejunum.

## Conclusions

This is a case report of rare late complications of side-to-side pancreaticojejunostomy; PR procedure. Re-anastomosis would be a treatment option for this rare complication.
